# OP Pesticides, Organic Diets, and Children’s Health: Lu et al. Respond

**Published:** 2006-10

**Authors:** Chensheng Lu, Richard A. Fenske, Dana B. Barr

**Affiliations:** Department of Environmental Health, Rollins School of Public Health, Emory University, Atlanta, Georgia, E-mail: clu2@sph.emory.edu; Department of Environmental and Occupational Health Sciences, University of Washington, Seattle, Washington; National Center for Environmental Health, Centers for Disease Control and Prevention, Atlanta, Georgia

Krieger et al. criticize the misrepresentation of our recent paper (Lu et al. 2006) with respect to the relevance to health risk reduction of dietary organophosphorus (OP) pesticide exposure in children. They argue that current OP exposures, measured in the form of urinary metabolites in children, are well below the “safe” level and therefore pose “zero” risk.

The basis for Krieger et al.’s extraordinary statement is the claim that “specific health risks” have never been associated with dietary pesticide exposures, and that “zero cases of disease” have occurred that can be attributed to such exposures. However, Krieger et al. must be aware of the tragic misapplication of the carbamate insecticide aldicarb to watermelons in California in 1986, resulting in 6 deaths, 17 hospitalizations, and > 1,000 probable or possible poisoning cases (Centers for Disease Control and Prevention 1986). The probability of such an event occurring again is certainly greater than zero. In fact, such an event was reported recently in Taiwan for an OP pesticide found in vegetables (Wu et al. 2001). Krieger et al. also ignore the fact that some pesticides are categorized as carcinogens and that dietary exposures to these compounds carry some risk. For example, the fungicide chlorothalonil is classified by the State of California as a carcinogen [Office of Environmental Health Hazard Assessment (OEHHA) 2006], and the U.S. Environmental Protection Agency (EPA) estimated that the cancer risk from dietary exposure to chlorothalonil is 1.2 × 10^−6^ (U.S. EPA 1999). Although one might agree with the U.S. EPA that this is a *de minimus* risk, the risk cannot be characterized as “zero.”

Krieger et al. appear to dismiss the possibility that pesticides can produce non-acute adverse health effects, but recent studies have shown an association between adverse neurologic and growth outcomes in children exposed to OP pesticides *in utero* (Jacobson and Jacobson 2006; Whyatt et al. 2005; Young et al. 2005). To our knowledge, no epidemiologic studies of children’s dietary OP pesticide exposures and adverse health effects have ever been conducted. To quote our current Secretary of Defense, Donald Rumsfeld, “Absence of evidence is not necessarily the evidence of absence” (Rumsfeld 2003). A final judgment of the potential for OP pesticide exposure to cause adverse developmental or neurologic health effects in children will require rigorous epidemiologic studies that include sound exposure assessment.

Risk is a probabilistic concept and is generally considered to be dependent on exposure and toxicity. If exposure is reduced, then the corresponding risk is reduced. We believe that the jury is still out on the risk, particularly on the chronic neurologic health risk in young children. In our article (Lu et al. 2006) we raised the hypothesis that by reducing children’s dietary exposure to OP pesticides, the risk of the associated health effects may be reduced. We look forward to future scientific evidence sufficient to either accept or reject this hypothesis. If our article has heightened unnecessary anxiety and fear among the public, this was not our intent. However, the perception of risk in the world of public health depends on individual attitudes and beliefs. Krieger et al. have misinterpreted our conclusion (Lu et al. 2006) as much as they have misunderstood the enforcement of the speeding limit, which is obviously not to issue citations to parked cars, but rather to minimize the possibilities of automobile accidents. The relevance of health risk reduction of dietary OP exposure in children is analogous to many public health campaigns in this county, such as the use of seat belts, smoking cessation, and HIV (human immunodeficiency virus) prevention, which are not adopted to penalize or inconvenience individuals, but are intended for public health protection.
